# Nonischemic Super-Responders in Fusion CRT Pacing with Normal Atrioventricular Conduction

**DOI:** 10.3390/diagnostics12092032

**Published:** 2022-08-23

**Authors:** Emilia-Violeta Goanță, Constantin-Tudor Luca, Cristina Vacarescu, Simina Crișan, Lucian Petrescu, Radu Vatasescu, Mihai-Andrei Lazăr, Andra Gurgu, Vladiana-Romina Turi, Dragos Cozma

**Affiliations:** 1 Cardiology Department, “Victor Babes” University of Medicine and Pharmacy, 2 Eftimie Murgu Sq., 300041 Timisoara, Romania; 2 Research Center of the Institute of Cardiovascular Diseases Timisoara, 13A Gheorghe Adam Street, 300310 Timisoara, Romania; 3 Institute of Cardiovascular Diseases Timisoara, 13A Gheorghe Adam Street, 300310 Timisoara, Romania; 4 Department of Cardiology, University of Medicine and Pharmacy “Carol Davila”, 014451 Bucharest, Romania; 5 Clinical Emergency Hospital, 014451 Bucharest, Romania

**Keywords:** fusion CRT pacing, LV-only pacing, super-responders

## Abstract

**Background**: Fusion CRT pacing (FCRT) is noninferior to biventricular pacing, according to the current data. The aim of this study is to assess the response to FCRT and to identify predictors of super-responders (*SRs)* in a nonischemic population with normal AV conduction. **Methods**: LV-only CRT patients (pts) with a right atrium/left ventricle pacing system implanted in two CRT centers in Romania were included. Device interrogation, exercise tests, echocardiography, and individualized drug optimization were performed every 6 months during close follow-up. SRs pts were defined as those with left ventricular end-systolic volume (LVESV) improvement ≥30% and stable ejection fraction (LVEF) ≥45%. **Results**: A total of 25 out of 83 pts (31%) were SRs, with nonischemic LBBB low EF cardiomyopathy (50 male, 62 ± 9 y.o.) initially included. Mean follow-up was 5 years ± 27 months. Patients were divided in two groups: SRs and non-SRs (52 responders/6 hypo-responders). Two predictors were found in the SRs group: a higher baseline LVEF (SRs 29 ± 5% vs. non-SRs 26 ± 5%, *p* = 0.02) and a lower pulmonary arterial systolic pressure (SRs 38 ± 11 mm Hg vs. non-SRs 50 ± 15 mmHg, *p* = 0.003). Baseline severe mitral regurgitation was found in 11% of SRs vs. 64% in the non-SRs group. **Conclusions**: SRs in the selected NICM-FCRT group are significative high. Higher baseline LVEF and mild pulmonary arterial hypertension were independently associated with super-response.

## 1. Introduction

Left Ventricular (LV) only pacing is a noninferior alternative to biventricular pacing (BiV) in patients undergoing cardiac resynchronization therapy (CRT) as a first-line treatment for symptomatic heart failure (HF), reduced ejection fraction (EF), and QRS duration >130 ms [1].

The necessity and position of an RV lead to deliver pacing to obtain better responsiveness to resynchronisation is highly debatable. The only need to stimulate pacing through an RV lead may be in the case of AV block and permanent atrial fibrillation (AF). LV-only pacing is analogous to fusion pacing, and it provides a similar outcome in patients with moderate to severe HF CRT [2,3]. Fusion pacing CRT (FCRT-p) is defined as optimized LV-only pacing with electrical and mechanical fusion with the intrinsic QRS and may substantially increase the structural response rate, likely by shortening LV activation time [4,5]. Recent data concerning FCRT-p without, an RV lead, also unveiled a positive outcome in nonischemic patients with normal AV conduction [6,7]. 

The AV conduction variability still remains the main concern regarding the quality of LV-only fusion pacing, both in the short and long term, due to changes in disease state, exercise capacity, and medication. The algorithm described by the investigators of the adaptive-CRT trial, which promotes intrinsic conduction and reduces RV pacing, demonstrated a high percentage of fusion pacing in patients with normal AV conduction, which was independently associated with superior clinical outcomes [8].

The rate of non-responders to CRT is still around 30%, although the advance of resynchronization is extensively studied [9,10]. Heterogenous response to CRT as an intuitive assumption may be due to mixing a nonhomogeneous population of ischemic and non-ischemic patients, with or without normal AV conduction or sinus rhythm/AF. One size does not fit all, as the same CRT approach cannot provide comparable and reproducible outcomes in diverse patients. 

The aim of this study is to assess the response to fusion pacing CRT (FPCRT) in a homogeneous nonischemic population with normal AV conduction, which also allows for true FPCRT. 

## 2. Materials and Methods

### 2.1. Inclusion and Exclusion Criteria 

This is a multicenter, retrospective study which included and analyzed patients between April 2011–June 2021. The study population included consecutive patients with CRT indication, dilated cardiomyopathy, New York Heart Association (NYHA) class II–III heart failure, left-ventricular ejection fraction (LVEF) ≤35%, QRS complex ≥130 ms, typical LBBB pattern, and optimal pharmacological treatment 3 months prior to CRT. Patients were excluded if any of the following were present: acute coronary syndrome or history of coronary artery disease, permanent atrial fibrillation, severe comorbidities (e.g., renal, lung, or liver failure, cerebral insufficiency, or terminal cancer), other causes of non-ischemic cardiomyopathy (sarcoidosis, dilated hypertrophic cardiomyopathy), evidence of conditions with risk of life-threating ventricular arrhythmias (family history of sudden cardiac death, ventricular tachyarrhythmias identified on Holter monitoring, extended fibrosis seen on MRI, and genetic abnormalities), or noncardiac diseases that limit physical activity (e.g., orthopedic conditions).

All patients were on chronic optimal medical therapy, including novel drug therapy for heart failure (Angiotensin Receptor-Neprilysin Inhibitor, sodium-glucose cotransporter-2 inhibitors) according to the current HF guidelines [11], individualized according to clinical and paraclinical variables (blood pressure, heart rate, renal function). 

### 2.2. Device Programming 

The strategy of device management followed precise steps: 1. Interrogation immediately after implant procedure; 2. A 24 h check after implantation; 3. A discharge check; 4. Programming at each follow-up visit. The following steps were performed in all patients after CRT: 1. A 12-lead ECG pacing on/off and complete interrogation. 2. All pacemakers were initially programmed at a base heart rate of 60 bpm and maximum tracking rate (MTR) of 130 bpm. 3. Check-ups included evaluation of the spontaneous AV interval, percentage of ventricular pacing, LV threshold, and adequate response of cardiac devices functioning during exercise. 4. The AV interval was programmed individually to achieve the best fusion capture. 

**Figure 1:**
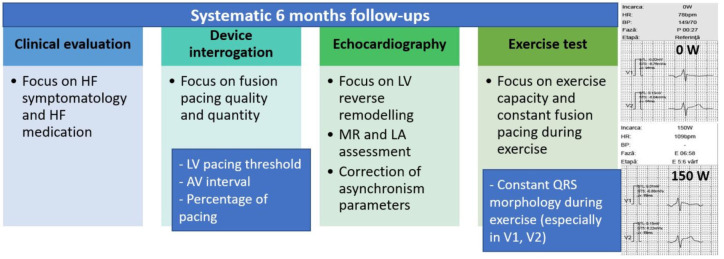
Follow-up evaluation algorithm in patients with fusion pacing CRT.2.3; responder and super-responder parameters.

The CRT assessment during exercise test included: maximal heart rate, beat-to-beat ECG analysis of true LV fusion pacing, loss of LV capture occurrence, and improvement of exercise capacity. In the case of LV loss of capture or unsatisfactory LV fusion pacing, reprogramming was performed individualized for each patient, and BB/ivabradine dose titration was performed to achieve stability of the PR spontaneous interval [12]. The following device optimizations were performed accordingly to the results of the exercise test: reprogramming of the dynamic AV interval, and reprogramming of the maximum tracking rate and rate response function. The algorithm presented in Figure 1 summarizes the main evaluation steps in our systematic follow-ups. 

The response assessment to CRT was based on the following criteria [12,13]: clinical response to CRT, defined as improvement in the NYHA functional class, ET duration, and workload; echocardiographic response (defined as >5% increase in LVEF, 15% decrease in LV end-systolic/diastolic volume and decreased mitral regurgitation degree); and assessment of outcomes, defined as the number of hospitalizations due to worsening heart failure, all-cause mortality, and morbidity.

Patients were divided in two groups: super-responders (SRs) and non-SRs (responders and hypo-responders). Pts were defined as non-SRs-subtype responders when a decrease in LVESV of ≥15% and absolute increase of >5% in LVEF at 6 months was found. Non-SRs-subtype hypo-responders were defined when there was no echocardiographic improvement or a decrease in LVESV of <15% and an absolute increase of <5% in LVEF at 6 months was found. SRs were defined as a 2-fold or greater improvement in LVEF or a final LVEF ≥ 45% at 6 months post-CRT. 

### 2.3. Echocardiographyc Evaluation

Transthoracic echocardiography (TTE) was performed in all patients. Ultrasound images were explored with patients in the left lateral decubitus position, using a VIVID 9 system (GE Health Medical, Milwaukee, WI, USA). An ECG was simultaneously recorded for each patient. The echocardiographic examination was performed using standard views and techniques. LV wall thickness, LV end-diastolic and end-systolic diameters (LVEDD, LVESD) and volumes (LVEDV, LVESV), LV ejection fraction (LVEF), LA diameter (LAd), LA area (LAA), and LA volume (LAV) were measured. Atrioventricular, interventricular, and intra-LV synchrony parameters were also assessed in all patients. All LA measurements were performed at the end-systole, just before mitral valve opening, and maximal atrium size was considered for evaluation. A complete TTE evaluation for CRT was performed in all patients.

### 2.4. Statisical Analysis 

Data are expressed as mean ± standard deviation for continuous variables and as proportions for categorical variables. Continuous variables were compared between groups using the unpaired *t* test (variables with normal distribution) or the Mann–Whitney U test (non-normally distributed variables). Proportions were compared using the chi-squared test and Fischer’s exact test. A *p* value < 0.05 was considered significant. To determine the predictors for super-responders to CRT, we employed univariate and multivariate analyses using logistic linear regression All analyses were carried out with the SPSS, version 18.0 (SPSS Inc., Chicago, IL, USA) statistical software.

## 3. Results

A total of 83 patients, 62.3 ± 9.2 y.o. (60% males), with non-ischemic dilated cardiomyopathy, HF NYHA class II-III, and CRT-P indication were included. All patients were implanted with a RA/LV pacing system between 2011–2021. All patients had a QRS complex > 130 ms with typical LBBB morphology (Strauss criteria). A comparison between the SR group and non-SR group regarding the main demographic parameters and medical treatments is found in Table 1. 

No major complications were noted intra- or periprocedural during implantation. At baseline, all devices were programmed at a resting rate of 50 beats/min and a maximum tracking rate (MTR) of 130 beats/min. Individualized AV interval programming with an AV pace of 148 ± 21 ms and an AV sense of 119 ± 25 ms, allowed fusion pacing in all patients. 

A comparison regarding technical aspects and post-CRT ECG in the SR and non-SR groups are presented in Table 2.

The anterolateral position was predominant in the non-SR group, while the SR group had a mainly posterolateral position of the LV lead. These technical aspects were determined by the anatomy of the patients. Taking into account that there were no anterior leads, only an anterolateral position, we can assume that lead position or post-CRT ECG had no major implications regarding the outcome in our population. 

The average EF at baseline was 26.7 ± 5.1%. All patients had severe LV dilatation (mean LVEDV 245.7 ± 86.8 mL) associated with important LA dilatation (LAV 101.9 ± 33.1 mL) and pulmonary hypertension (sPAP 46.1 ± 15.3 mmHg). Echocardiography data are presented in Table 3. Severe mitral regurgitation (MR), at baseline, was found in 50% of patients, 40% of patients had moderate MR, and 10% had mild MR. 

The average follow-up was 5 years ± 27 months (median value 3.2 years, with a range between 11 years and 1 year). A total of 31 patients (40%) were implanted before 2016 and have had more than 5 years of follow-up. At the end of the follow-up, NYHA class improvement was noted in 87% of patients: 38% of patients in NYHA I, 53% in NYHA II (versus 51% at baseline), 9% in NYHA III (versus 49% at baseline). 

**Figure 2:**
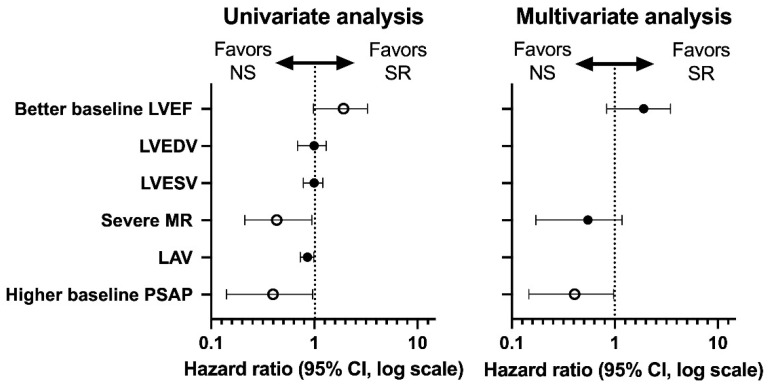
Univariate and multivariate analysis of the echocardiographic parameters. The empty circles represent the statistically significant difference.

The univariate analysis of the echocardiographic parameters showed that a better LVEF at baseline (HR = 1.917; 95% CI 0.983 to 3.271) were predictors for SR, while severe MR (HR = 0.431; 95% CI 0.212 to 0.943) and a higher PSAP (HR = 0.398; 95% CI 0.141 to 0.959) were predictors for NS. These three parameters were included in the multivariate analysis, and only a higher PSAP at baseline (HR = 0.406; 95% CI 0.146 to 0.971) remained an independent predictor for NS. A forest plot graph with univariate and multivariate analysis (hazard ratio and 95% confidence interval—CI, log scale) is shown in Figure 2.

**Figure 3:**
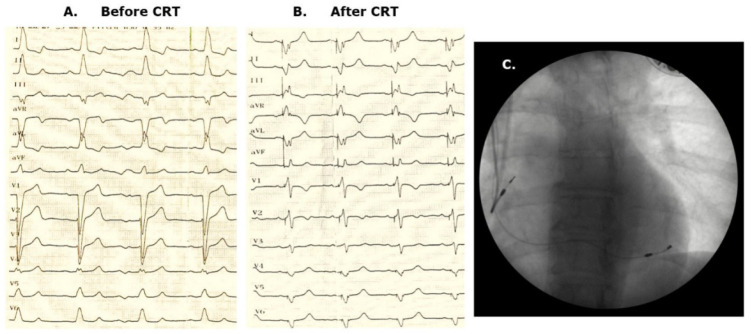
(**A**)**.** ECG before CRT—typical LBBB morphology with a QRS of 160 ms. (**B**). ECG after fusion CRT-P: a thinner QRS can be easily noted, with an rS morphology in V1 and V2, and a QS morphology in the left leads. (**C**)**.** Posterior-anterior fluoroscopy image: CRT DDD pacing system with 2 leads in the right atrium and a lateral branch of the coronary sinus.

The first patient implanted with a RA/LV DDD device has 10 years of follow-up, and he is a super-responder. An obvious super-responder profile, with a ”beautiful” fusion pacing ECG (Figure 3A–C) was noticed at the 3-month follow-up (LV vol 170 mL, EF 45–50%); the exercise test, echocardio testing, and device fine tuning were systematically performed every 6 months, with zero hospitalization for worsening HF. The device was changed after 9 years. In such a long follow-up, the patient had a normal life, with predictable weight gain according to the national trend evolution for a ”modern society,” and a new treatment for type 2 diabetes mellitus was initiated.

The exercise test was an essential method of follow-up; more than 400 ET were performed in order to ensure adequate fusion pacing CRT in all patients, both at rest and during exercise. The following interventions were done to achieve 100% fusion pacing: reprogramming sensed/paced AV delay or rate adaptive AV (beat-to-beat ECG analysis was performed during all stages of exercise), increasing maximum tracking rate (MTR) because of inadequate or lost LV capture, and programming rate modulation response due to chronotropic incompetence. Device optimization was performed in 32 pts (38%), while BB and ivabradine titration to maintain and stabilize the AV interval was performed in more than 170 of the ETs. A similar number of optimizations (regarding the number of patients) were performed in the SR and non-SR groups, with an increased exercise capacity in the SR group. Specific data regarding the exercise test parameters are shown in Table 4.

Non-sudden cardiac death occurred in 7 patients (9%). No deaths were recorded in the super-responders group. Readmissions due to worsening heart failure were noted in 20 patients (24%): A total of 8 patients due to atrial flutter or fibrillation, and 12 patients due to medication and diet issues. Pacemaker replacement was performed in 6 patients due to an elective replacement indicator.

A CRT-D upgrade was not necessary in any of our patients. Amiodarone was introduced for paroxysmal AF episodes (diagnosed at device interrogation) and persistent AF in 12 patients (14%). Direct current cardioversion for persistent AF was required in 6 patients (7%). Typical atrial flutter cavotricuspid ablation was performed in 2 patient (2%). There were 3 patients treated with betablocker and amiodarone who developed a subsequent AV block and needed an upgrade to a triple-chamber device (classical CRT-P). The final outcomes were positive, without any other complications in these patients. 

## 4. Discussion

There are three categories of CRT response definition. The first category includes clinical measures: NYHA class, quality of life, 6 min walk test, and exercise duration. The second category is based on LV reverse remodeling assesment: increase in LV ejection fraction (LVEF), reduction in LV end systolic and dyastolic volumes (LVESV, LVEDV), and mitral regurgitation (MR). The third category measures the outcome regarding HF hospitalizations, morbidity, and all-cause mortality reduction. A CRT evaluation in clinical trials is different from those in real life. We must manage CRT candidates without adequate evidence or consensus of opinion in LBBB nonischemic cardiomyopathy. Inappropriate selection of the CRT patients in clinical trials always raises the same questions (CRT-p or CRT-d; superiority of a specific location for the RV lead, i.e., septal, outflow tract or apical; programming of the device post-CRT-LV pacing or post-biventricular pacing). Due to these ambiguities, endpoints are very different in clinical trials and in daily practice. These issues should be addressed in future clinical research; perhaps genetic testing and cardiac MRI in these patients could help to assess the risk of malignant arrhythmias and choose the proper device. If a CRT-p device is needed, do we still need an RV lead, or is an LV-only lead is enough, with proper fusion pacing? Some of these issues are addressed in our study using a highly selected NICM LBBB group.

The most reliable echocardiographic parameters to use are LVEF and LVES. Responder patients are defined if they show a decrease in LVESV of ≥15%, or an absolute increase of >5% in LVEF at the 6-month follow-up visit compared with the baseline echocardiogram (11). They are labeled as super-responders (SRs) if they show a 2-fold or greater improvement in LVEF or a final LVEF ≥ 45% at 12 months post-implantation [13,14].

Our study addresses a homogeneous group of highly selected NICM with long-term follow-ups. We know that NICM is a heterogeneous condition, and the reversibility of systolic dysfunction and overall prognosis is related to etiology (ischemic vs. nonischemic), life-shortening co-morbidity (e.g., advanced chronic kidney disease or severe lung disease), patient selection (typical LBBB-Strauss criteria vs. atypically LBBB), severe symptoms (NYHA IV), and proper follow-up (treatment optimization, echo and device reprograming). In our study population, patients with severe mitral regurgitation and pulmonary hypertension at baseline had a low chance of becoming super-responders. The small number of patients included in this analysis represents the main limitation of this study. In a paper published by H. Burri et al., on behalf of the EHRA Education Committee, LV-only pacing was associated with a better CRT outcome and a decrease in the number of non-responders [15]; however, few data exist regarding fusion pacing super-response, and even fewer for CRT without an RV lead. LV-only pacing can be performed if AV conduction is normal in classical CRTP, while fusion pacing CRT without an RV lead is feasible if the patients have no indication for an RV lead (absence of CRTD indication). 

The population included in our study was divided in two groups: super-responders (SRs) and non-SRs (responders and hypo-responders). From the non-SRs (69%) group, 52 were responders and 6 were considered hypo-responders at 6 months after CRT. In general, our population had a very positive outcome to FCRT, with more than one-third being SRs (31%). This can be explained due to strict enrollment criterias (nonichemic etiology, typical LBBB, absence of severe comorbidity and less severe symptoms, NYHA II-III) of patients, with a high probability of respondes to CRT in the longer term. No arrhythmic deaths were recorded in this population. Indeed, new medications, such as ARNI and SGLT2I, may influence the outcome in HF patients. Nevertheless, this study analyzed the CRT response and at 6 months follow-up, all the patients were on optimal medical treatment at the moment of implantation and up to the 6 month follow-up. Being on stable medication at baseline and at 6 months follow-up decreases the chances of a possible interference with the CRT response, with the observation that we noted the most significant improvement at 6 months post CRT. On the other hand, on long-term follow-up, we are confident that medication optimization plays a key role in maximazing CRT response. Drugs such as betablockers (BBs) and ivabradine were titrated according to exercise test results in order to maintain long-term constant fusion pacing [12]; the conclusion of this study was that BBs/ivabradine titration and routine ET during follow-ups in patients with fusion CRTP should be a standard approach to maximize the resynchronization response. 

After the controversial Danish trial [16], the ICD benefit in primary prophylaxis in patients with non-ischemic cardiomyopathy is debatable in the presence of CRT; moreover, a post-CRT value well above 40%—or even close to 50%—may imply that the subsequent risk of sudden cardiac death will be low.

Based on the results of a meta-analysis [17], the use of concomitant CRT appears to impact the benefit attained by ICDs, with no significant reduction in mortality. These results are consistent with another analysis, which showed that although combined CRT-ICD decreased mortality compared to control, it did not result in further improvement in survival compared to CRT therapy alone [18]. CRT results using reverse remodeling showed a decrease in the risk of ventricular arrhythmias [19,20], attenuating the effect of ICD. Furthermore, it has been shown that a non-ischemic etiology is a prognostic factor for favorable response to CRT [21,22]. 

The individual programming of devices after CRT implantation and regular follow-up should be the aim. The key target of programming has been to deliver 100% of ventricular capture in order to achieve the maximal outcomes [1,23]. Although there is no randomized trial comparing a lower vs. a higher degree of ventricular pacing, observational data state that a low degree of ventricular pacing is associated with poor outcome.

Variability of AV conduction is one of the main concerns regarding proper FCRT at rest and during exercise. Our study shows the importance of periodic follow-up (including TTE and ET) in order to maximize the percentage of responders. To ensure the stability of AV conduction in our population, we used drug optimization and CRT reprogramming during follow-ups with ET. The ET check was an essential method of follow-up, performed systematically in our series with more than 400 ETs to ensure adequate fusion pacing CRT in all patients. Device optimization was needed in more than one-third of the patients, while BB and ivabradine titration was performed in almost half of ETs. 

Poor reprogramming of AV delays is a contributor to reduced efficacy of CRT [24], as we already know. Post-CRT echocardiography, with assessment of the mitral inflow pattern, allows for a quick evaluation of the appropriateness of the AV-interval programming. The Adaptive-CRT trial, which promotes fusion pacing at a heart rate < 100 b/min, randomized 522 patients in a 2:1 ratio to CRT optimization with the AdaptivCRT™ algorithm vs. echocardiography [25]. In a sub-analysis of the study [8], patients with ≥50% of synchronized LV pacing showed lower hospitalization for worsening of heart failure and mortality compared with those with <50% synchronized LV pacing. In patients with normal AV conduction, the algorithm significantly increased the rate of CRT response compared with the echo arm and lowered the risk of death or heart failure hospitalization. Data on the AdaptivCRT™ algorithm for FCRT are promising, but validation is needed. In our cohort of patients, we have provided exclusive fusion pacing CRT using RA/LV dual chamber devices, without the potential of additional desynchronization through right ventricular pacing.

An ongoing trial is expected, as AdaptResponse (NCT02205359) set out to recruit approximately 3000 patients with LBBB, normal AV conduction, and LV fusion pacing with a combined primary endpoint of all-cause mortality and heart failure decompensation [26]. We will definitely obtain more solid data for FCRT from this trial. The ideal algorithm would be the one that automatically updates AV intervals to accommodate changes in AV delay and to ensure the optimal fusion between the activation wavefronts originating from intrinsic conduction and LV pacing electrode at all times. 

## 5. Conclusions

Fusion CRT pacing shows a positive outcome in a highly selected NICM group, with over one-third of patients being SRs after 6 months. Regular follow-ups (including ET, echocardiography, device reprogramming, and treatment optimization) maximize the percentage of CRT responders.

## Figures and Tables

**Table 1:** The main demographic parameters and medical treatments in all patients and groups of SRs and non-SRs.

		All Patients (N = 83)	Super-Responders (N = 25, 31%)	Non-SRs (N = 58, 69%)	*p* *
Male gender, %		50 (60%)	17 (65%)	33 (57%)	-
Age, y.o, mean ± SD		62.3 ± 9.2	60.9 ± 11.2	63.2 ± 8.7	0.3746
HF, N, %	NYHA II	42 (51%)	21 (50%)	21 (51%)	-
	NYHA III	41 (49%)	21 (50%)	20 (49%)	-
Associated pathology, N, %	Hypertension	34 (41%)	13 (50%)	21 (36%)	-
	CKD	39 (47%)	9 (35%)	30 (52%)	-
	Diabetes Mellitus	34 (41%)	10 (40%)	24 (41%)	-
	COPD	22 (26%)	5 (20%)	17 (30%)	-
QRS duration, ms, mean ± SD		160.6 ± 16	162.2 ± 13.8	159.9 ± 17	0.5979
PR interval, ms, mean ± SD		186.7 ± 32.4	189.3 ± 33.5	185.2 ± 33.2	0.6495
PR interval, ms, range of values		120–240	140–240	120–220	-
Medical treatment, N, %	Bblockers	76 (91%)	19 (75%)	57 (98%)	-
	ACEI/ARB	71 (86%)	21 (85%)	50 (86%)	-
	Antialdosteronics	72 (87%)	19 (75%)	54 (93%)	-
	ARB + ARNI	9 (11%)	3 (10%)	6.4 (11%)	-
	SGTL2	10 (12%)	4 (16%)	6 (10%)	-
	Ivabradine	43 (52%)	11 (45%)	32 (55%)	-

N—number of patients; CKD—chronic kidney disease; COPD—chronic obstructive pulmonary disease; SD—standard deviation; ACEI—angiotensin converting enzyme inhibitor; ARB—angiotensin receptor blockers; ARNI—angiotensin receptor neprilysin inhibitor; SGTL2—sodium-glucose cotransporter-2 inhibitors. * Value of *p* calculated for SR and non-SR group.

**Table 2:** Technical aspects in the SR and non-SR groups.

		Super-Responders (N = 25, 31%)	Non-SRs (N = 58, 69%)	*p*
LV lead position N, %	Posterolateral	9 (35%)	14 (24%)	-
	Lateral	6 (25%)	16 (27%)	-
	Posterior	3 (10%)	10 (18%)	-
	Anterolateral	5 (20%)	17 (30%)	-
	Epicardial	2 (8%)	1 (2%)	-
AV paced interval, mean ± SD		147.5 ± 17.3	149.2 ± 22.1	0.7623
AV sensed interval, mean ± SD		113.3 ± 22.2	122.9 ± 26.2	0.1559
Post CRT ECG	Rs in V1, V2	23 (90%)	51 (88%)	-
	Qs/qS in D1, avL	20 (80%)	41 (70%)	-
	QS/qS in V5,V6	18 (70%)	47 (57%)	-

**Table 3:** Echocardiography parameters in SR versus non-SR groups.

	SR (N = 25, 31%)	Non-SR (N = 58, 69%)	*p*
LVEF (%) - Baseline - 6 months follow-up - Relative change, %	28.7 ± 4.9 43.6 ± 3.9 35%	25.6 ± 4.8 30 ± 5.1 15%	**0.0204** - -
LVEDV, mL, mean ± SD - Baseline - 6 months follow-up - Relative change, %	237.4 ± 118.2 168.4 ± 69.1 30%	248.17 ± 67.9 230.8 ± 80.4 7%	0.6458 - -
LVESV, mL, mean ± SD - Baseline - 6 months follow-up - Relative change, %	204.4 ± 117.8 95.5 ± 55.1 54%	183 ± 58.2 137.1 ± 44.2 26%	0.3325 - -
Severe MR, n, % - Baseline - 6 months follow-up	3 (11%) 1 (5%)	38 (64%) 18 (31%)	0.0410 -
LAV, mL, mean ± SD - Baseline - 6 months follow-up - Relative change, %	92.8 ± 30.6 81.5 ± 25.5 13%	105.5 ± 33.4 103.9 ± 40.6 2%	0.1532 - -
PSAP, mmHg, mean ± SD - Baseline - 6 months follow-up - Relative change, %	38 ± 11.5 30.4 ± 7.3 20%	49.6 ± 15.3 47.2 ± 12.3 5%	0.0037 - -

**Table 4:** Exercise test main parameters and interventions for device and medication optimization.

ET Parameters and Interventions	SR (N = 25, 31%)	Non SR (N = 58, 69%)	*p*
Exercise duration, min	8 ± 3.4	7 ± 2.8	0.1660
Exercise load, W	119 ± 23	98 ± 27	0.0011
Exercise capacity, METS	6.5 ± 1.4	5.3 ± 1.2	0.0002
Device programming, N, %	13 (52%)	19 (33%)	-
Changes in medication (betablokers and ivabradine titration), N, %	15 (60%)	17 (29%)	-

METS = metabolic equivalents of task; min = minute; N = number of patients; W = watts.
